# Diabetes-Induced Autophagy Dysregulation Engenders Testicular Impairment via Oxidative Stress

**DOI:** 10.1155/2023/4365895

**Published:** 2023-02-03

**Authors:** Renfeng Xu, Fan Wang, Zhenghong Zhang, Yan Zhang, Yedong Tang, Jingjing Bi, Congjian Shi, Defan Wang, Hongqin Yang, Zhengchao Wang, Zonghao Tang

**Affiliations:** ^1^Key Laboratory of Optoelectronic Science and Technology for Medicine of Ministry of Education, Provincial Key Laboratory for Developmental Biology and Neurosciences, College of Life Sciences, Fujian Normal University, Fuzhou 350007, China; ^2^Provincial University Key Laboratory of Sport and Health Science, School of Physical Education and Sport Sciences, Fujian Normal University, Fuzhou 350007, China; ^3^Fujian Provincial Key Laboratory of Reproductive Health Research, School of Medicine, Xiamen University, Xiamen 361102, China; ^4^Drug Discovery Research Center, Key Laboratory of Medical Electrophysiology, Ministry of Education, Department of Pharmacology, School of Pharmacy, Southwest Medical University, Luzhou 646000, China; ^5^Cedars-Sinai Medical Center, Los Angeles 90048, USA

## Abstract

Testes produce sperms, and gamete generation relies on a proper niche environment. The disruption of hierarchical regulatory homeostasis in Leydig or Sertoli cells may evoke a sterile phenotype in humans. In this study, we recapitulated type 2 diabetes mellitus by using a high-fat diet- (HFD-) fed mouse model to identify the phenotype and potential mechanism of diabetes-induced testicular impairment. At the end of the study, blood glucose levels, testosterone structure, testicular antioxidant capacity, and testosterone level and the expression of hypoxia-inducible factor- (HIF-) 1*α*, apoptosis-related protein cleaved-caspase3, and autophagy-related proteins such as LC3I/II, p62, and Beclin1 were evaluated. We found that long-term HFD treatment causes the development of diabetes mellitus, implicating increased serum glucose level, cell apoptosis, and testicular atrophy (*P* < 0.05 vs. Ctrl). Mechanistically, the results showed enhanced expression of HIF-1*α* in both Sertoli and Leydig cells (*P* < 0.05 vs. Ctrl). Advanced glycation end products (AGEs) were demonstrated to be a potential factor leading to HIF-1*α* upregulation in both cell types. In Sertoli cells, high glucose treatment had minor effects on Sertoli cell autophagy. However, AGE treatment stagnated the autophagy flux and escalated cell apoptosis (*P* < 0.05 vs. Ctrl+Ctrl). In Leydig cells, high glucose treatment was adequate to encumber autophagy induction and enhance oxidative stress. Similarly, AGE treatment facilitated HIF-1*α* expression and hampered testosterone production (*P* < 0.05 vs. Ctrl+Ctrl). Overall, these findings highlight the dual effects of diabetes on autophagy regulation in Sertoli and Leydig cells while imposing oxidative stress in both cell types. Furthermore, the upregulation of HIF-1*α*, which could be triggered by AGE treatment, may negatively affect both cell types. Together, these findings will help us further understand the molecular mechanism of diabetes-induced autophagy dysregulation and testicular impairment, enriching the content of male reproductive biology in diabetic patients.

## 1. Introduction

The rising incidence of male infertility has become a global issue in recent decades. Like the increase in many cancer cases, the causes of this outcome are multifactorial, including workplace exposure [1], environmental change [2], drug intake [3], and lifestyle factors [4]. Among these, it has been accepted that the loss of dietary control and its derivate fat and type 2 diabetes mellitus (T2DM) are major factors that engender male infertility [5]. Even several studies have linked the association between T2DM and male infertility, but the underlying mechanisms remain unclear.

Testes are the sites where sperms are produced. The spermatogenic process requires the Sertoli cells, which nourish spermatocytes [6], and Leydig cells, which regulate the Sertoli cell function by producing testosterone [7]. Several studies have demonstrated that the disorder of metabolism in Sertoli cells and the increased apoptosis in Leydig cells contribute to the testicular structure alteration in diabetics [8, 9]. Oxidative stress contributes to the development of diabetic complications [10]. Simultaneously, oxidative stress is one of the leading causes of male infertility [11]. Numerous studies have shown that oxidative stress can induce blood-testis barrier (BTB) damage by mediating a series of intracellular signaling pathways, such as activating the p38/MAPK pathway [12, 13]. In addition, sperm are particularly susceptible to oxidative stress, with low levels of reactive oxygen species (ROS) playing an essential role in spermatogenesis. However, excess ROS can lead to oxidative damage to sperm, including reduced sperm concentration and motility and reduced fertilization [14]. Mammalian cells have developed several strategies to counteract ROS. On the one hand, ROS could be enzymatically quenched by superoxide dismutase (SOD), catalase (CAT), and glutathione peroxidase (GPX). On the other hand, ROS can be balanced by removing ROS origins, including impaired mitochondria and redundant protein aggregations, by autophagy [15, 16]. In the testes, autophagy plays an essential role in the Sertoli cell function, especially in the assembly of ectoplasmic specialization [17]. Similarly, autophagy is involved in testosterone production by facilitating cholesterol uptake [18]. It is noteworthy that abnormal regulation of autophagy is also a contributor to impaired testicular functions, and this phenomenon has been detected in several diabetic animal models [19–21], highlighting the potential role of autophagy in diabetes-induced testicular pathogenesis. However, many of these studies evaluated the change of autophagy levels at the whole testicular level and did not discriminate between different cell types in the testes, which may cause the unspecificity or inaccuracy of conclusions.

Autophagy, an evolutionarily conserved self-degradation mechanism, affects cellular metabolism and may help balance stress-induced functional disorders [22–24]. Autophagy levels vary in different statuses and could be regulated by many stresses or metabolism-related signaling pathways. For example, autophagy could be induced under serum disruption via Akt/mTOR pathway and hypoxia in a HIF-1*α*-dependent manner [25, 26]. Previous investigations have established that diabetic context can exacerbate the homeostasis of HIF-1*α* content in several organs of diabetic patients, which enables the progression of diabetic complications. In the male testes, an increase of HIF-1*α* is linked to the pathogenesis of varicocele [27]. HIF-1*α* is also necessarily required for the process of spermatogenesis [28]. Based on our knowledge, no studies have evaluated the role of HIF-1*α* in diabetes-induced testicular dysfunction. Furthermore, it is known that advanced glycation end products (AGEs) exert a regulatory role on HIF-1*α* expression in Leydig cells [29], but its pathological effects remain poorly understood.

In the present study, we constructed a high-fat diet-fed mouse model to understand the influences of diabetes on testicular morphology and implicated mechanisms, since previous studies have shown that diabetes can cause testicular damage, even infertility, but the underlying mechanism is not clear. The data showed that diabetes could drive the alteration of normal testicular morphology and disrupt the balance of cellular oxidative status. We also showed that the dimorphic manipulation of autophagic status in Sertoli and Leydig cells may drive the progression of testicular pathogenesis in diabetic mice. Collectively, these findings will contribute to our further understanding of the molecular mechanisms of diabetes-induced autophagy dysregulation and oxidative stress leading to testicular damage and further enrich the content of male reproductive biology in diabetic patients, providing a theoretical basis for subsequent studies.

## 2. Materials and Methods

### 2.1. Animals

All male C57/BL6J mice (4 weeks) were purchased from Wushi Experimental Animal Supply Co. Ltd. (Fuzhou, China). The animals were maintained under a 14 h and 10 h light-dark schedule with a continuous supply of chow and water. The high-fat diet (HFD) contained 58% lard oil, 25.6% carbohydrate, and 16.4% protein, while the normal control diet (NCD) contained 11.4% fat, 62.8% carbohydrate, and 25.8% protein [30]. The mice were randomly divided into two groups (*n* = 10/group) and were fed with either NCD or HFD for 8 weeks. The testes were then excised for further analysis. The Institutional Animal Care and Use Committee and the Ethics Committee on Animal Experimentation at Fujian Normal University approved the experimental protocol. All efforts were made to minimize animal discomfort and to reduce the number of animals used.

### 2.2. Immunofluorescent Staining

The testes were fixed in 10% neutral formalin for 48 h at 23°C ± 2°C. After fixation, these testes were embedded in paraffin, and 5 *μ*M sections were cut and mounted on slides. For immunofluorescent (IF) staining, the sections were dried, dewaxed, and rehydrated, followed by antigen retrieval with citrate buffer solution (pH 6.0). The sections were then incubated with anti-LC3 antibody (1 : 200 dilution) and anti-p62 antibody (1 : 200 dilution) overnight at 4°C. Goat serum (Boster Biological Technology, Wuhan) was used as negative control instead of a primary antibody. After washing with PBS, sections were incubated with secondary antibody (A20204 or A20206, Invitrogen, Carlsbad, CA) at RT for 1 h. Finally, the sections were counterstained with 4′,6-diamidino-2-phenylindole (DAPI) and mounted with coverslips.

### 2.3. Western Blotting

The testes were removed from mice with great care, followed by subcomponent isolation. Briefly, Leydig cells were isolated using a 70 *μ*M cell strainer after the disruption of the tunica albuginea. Seminiferous tubules were collected and minced and then subjected to EDTA (pH 7.4) treatment to remove peritubular cells to purify Sertoli cells. The tubular pellet was digested with collagenase for 10 min at 37°C to remove the germinal cells. The Sertoli cell suspension was collected by centrifugation at 300 × *g* for 5 min. The Leydig and Sertoli cells were lysed using ice-cold RIPA buffer supplemented with protease inhibitors (protease inhibitor cocktail, Beyotime Institute of Biotechnology, Haimen, SU) for protein extraction. Protein concentrations were determined using the BCA Kit (Beyotime Institute of Biotechnology, Haimen, SU) with bovine serum albumin standards. Subsequently, 40 *μ*g protein samples were loaded onto SDS-PAGE gel and separated by electrophoresis. The membranes were blocked by 5% skimmed milk, followed by primary antibody incubation at 4°C overnight (Supplementary Table 1). After washing, the membranes were incubated with horseradish peroxidase-conjugated goat antirabbit or mouse IgG (1 : 1000 dilution, Beyotime Institute of Biotechnology, Haimen, SU) for 1 h at RT. Eventually, the bands were visualized by enhanced chemiluminescence star (ECL, Beyotime Institute of Biotechnology, Haimen, SU). The bands were quantified using the ImageJ 1.49 software (National Institutes of Health, Bethesda, MD).

### 2.4. ROS Detection

For tissular detection, the testes were removed and dissociated according to the protocol in a previous study [31]. After centrifugation, the cells were homogenized in lysis buffer (250 mM sucrose, 20 mM HEPES-NaOH, pH 7.5, 10 mM KCl, 1.5 mM MgCl2, 1 mM EDTA, 1 mM EGTA, and protease cocktail inhibitor) [32], and the supernatants were collected by centrifugation at 10,000 × *g*, 4°C for 5 min. The mixes were suspended in HBSS containing dichlorodihydrofluorescein diacetate (DCFH-DA) (Jiancheng Biotech Institute, Nanjing, SU). Then, the mixes were incubated at 37°C for 30 min in the dark. The fluorescence of DCF was measured using a microplate reader at an excitation wavelength of 488 nM and an emission wavelength of 535 nM. The intracellular ROS level was measured using a commercial kit (Beyotime Institute of Biotechnology, Haimen, SU). Briefly, cells were washed with cold PBS and then incubated with DCFH-DA at 37°C for 20 min. Subsequently, cells were washed with PBS before measurement, and a microplate reader was used to determine the DCF fluorescence of 20,000 cells at an excitation wavelength of 488 nM and an emission wavelength of 535 nM.

### 2.5. Detection of Oxidative Stress Markers

SOD, GPX, and CAT were detected in testicular tissues using commercial kits (Jiancheng Bioengineering Institute, Nanjing, SU) according to the manufacturer's instructions.

### 2.6. Immature Sertoli Cell Culture

According to a previous study, three-week-old mice were used for Sertoli cell extraction [31]. Briefly, the testes were removed and decapsulated before Sertoli cell isolation. Then, the testes were digested with 0.1% collagenase (SCR103, Sigma) in HBSS (14025092, Gibco) for 10 min in a 37°C incubator. During digestion, the tube was gently shaken to promote tissue dispersing. The suspension was then filtered through a 70 *μ*M filter. Seminiferous tubules were collected and minced, followed by EDTA (pH 7.4) treatment to remove the peritubular cells. Afterward, the germinal cells were removed by digesting the tubular pellet with collagenase for 10 min at 37°C. The suspension of Sertoli cells was collected by sedimentation and then resuspended in a Sertoli cell culture medium (4521, ScienCell).

### 2.7. Mitochondria Isolation

The mitochondria were isolated using a commercial kit (C3601, Beyotime Institute of Biotechnology, Haimen, SU) to detect the level of cytosolic cytochrome C. Briefly, after the isolation, cells were washed with chilled PBS and then centrifuged at 600 × *g* to obtain the pellet. The pellet was resuspended in the isolation reagent with PMSF, followed by pipetting up and down several times and incubation on ice for 15 min. It was then centrifuged at 600 × *g*, 4°C for 10 min. Subsequently, the resulting pellet was transferred into a new tube and centrifuged at 10,000 × *g*, 4°C for 10 min. Finally, the supernatant was collected for Western blotting analysis.

### 2.8. Statistical Analysis

All data are presented as means ± SD. The significant differences within or between groups were evaluated by a one-way analysis of variance, followed by Tukey's multiple range test. Statistical analysis was conducted using SPSS version 20 software, and *P* < 0.05 was recognized as a statistically significant difference.

## 3. Results

### 3.1. Diabetes Induces Testicular Apoptosis and Structural Atrophy

**Figure 1:**
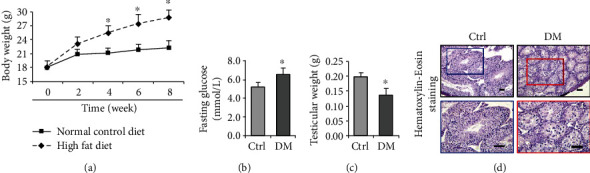
Changes of testicular weight and histology in diabetic mice induced by high-fat diet. (a) The growth curve of mice fed with normal control diet or high-fat diet. (b) Effect of diabetes on fasting glucose. (c) Effect of diabetes on testicular weight. (d) Effect of diabetes on testicular histology. The testicular morphology of diabetic mice was impaired, with disordered arrangement of spermatocytes observed by H&E staining. DM: diabetes mellitus. *P* < 0.05 was considered to indicate a statistically significant difference. Bar = 100 *μ*m. ^∗^*P* < 0.05 vs. Ctrl.

**Figure 2:**
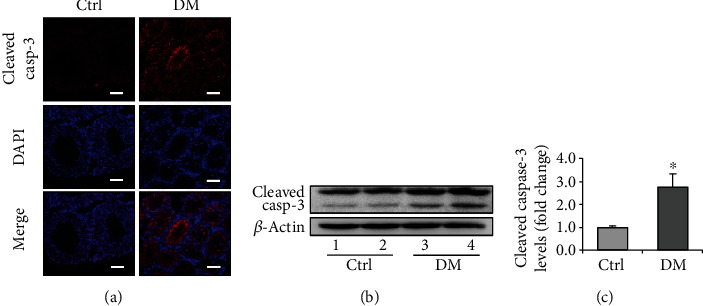
Expression of cell apoptosis marker, cleaved caspase-3, in the testis of diabetic mice. (a) The immunofluorescent staining of cleaved caspase-3 in the mouse testis. (b) Representative immunoblotting of cleaved caspase-3. (c) Densitometric qualification of cleaved caspase-3. DM: diabetes mellitus. *P* < 0.05 was considered to indicate a statistically significant difference. Bar = 100 *μ*m. ^∗^*P* < 0.05 vs. Ctrl.

To model T2DM, the mice were fed HFD for 8 weeks. Expectedly, HFD-treated mice showed T2DM phenotypes, including the fortified gain of body weight (Figure 1(a)) and escalated serum glucose (Figure 1(b)). In addition, the testes from HFD-treated mice exhibited diminished size and weight compared to those of the control, suggesting the atrophy of the testes (Figure 1(c)). Morphologically, the diabetic testes showed chaotic structural organization and curtailed size of seminiferous tubule (Figure 1(d)). We, therefore, wondered if diabetes drives testicular apoptosis and thus detected the expression of apoptotic marker cleaved caspase-3, which showed a palpable enhancement in diabetic mice (Figure 2(a)). This result was further validated by Western blotting (Figure 2(b)). These results suggested that HFD enabled the development of T2DM phenotype, accompanied by increased cellular apoptosis and atrophy of testicular structure.

### 3.2. Diabetes Has Dual Effects on Autophagy Regulation in Sertoli and Leydig Cells

**Figure 3:**
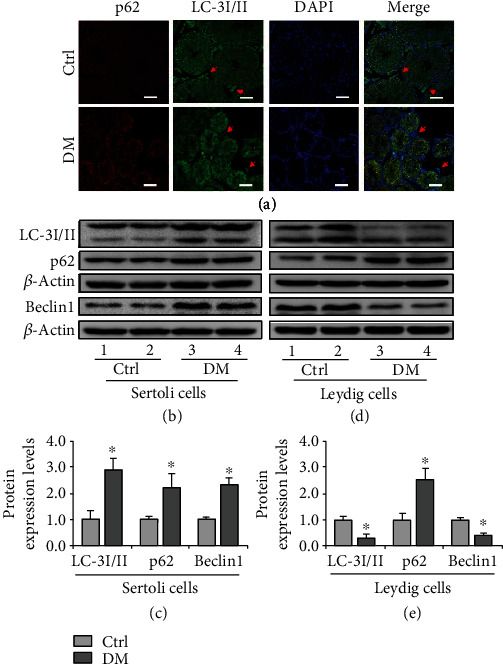
Expression of LC-3I/II, p62, and Beclin1 in the testicular cells *in vivo* and *in vitro*. (a) The immunofluorescent staining of p62 and LC-3I/II in the testis of diabetic mice. Red arrow indicates Leydig cells. (b) Representative immunoblotting of LC-3I/II, p62, and Beclin1 in Sertoli cells. (c) Densitometric qualification of LC-3II, p62, and Beclin1 in Sertoli cells. (d) Representative immunoblotting of LC-3I/II, p62, and Beclin1 in Leydig cells. (e) Densitometric quantification of LC-3II, p62, and Beclin1 in Leydig cells. Diabetes exerts dual effects on autophagy regulation in Sertoli and Leydig cells. DM: diabetes mellitus. *P* < 0.05 was considered to indicate a statistically significant difference. Bar = 100 *μ*m. ^∗^*P* < 0.05 vs. Ctrl.

As autophagy is a mechanism for ectoplasmic specialization in Sertoli cells [17] and steroidogenesis in Leydig cells [18], we next detected the autophagy-related markers LC-3I/II, p62, and Beclin1 by IF staining. Interestingly, the expression changes of LC-3I/II, p62, and Beclin1 exhibited divergent trends in Sertoli cells and Leydig cells. Briefly, the level of LC-3I/II and Beclin1 was increased in Sertoli cells while it decreased in Leydig cells (Figure 3(a)). Meanwhile, we noticed the concomitant increase of p62 in diabetic mice (Figure 3(a)), suggesting the disordered autophagy regulation in both cell types. The results of AO staining also confirmed this conclusion (Supplementary Figure 2). To further verify this result, we isolated Sertoli and Leydig cells and proved that our separation was successful by identifying the respective specific markers of Sertoli and Leydig cells (Supplementary Figure 1) and then detected the contents of LC-3I/II, p62, and Beclin1 by Western blotting. Consistently, we observed the augmentation of LC-3I/II, p62, and Beclin1 expression levels in Sertoli cells (Figures 3(b) and 3(c)) while decreased levels of LC-3II and Beclin1 in Leydig cells (Figures 3(d) and 3(e)). These findings indicated that diabetes exerts dimorphic roles during autophagy regulation in Sertoli and Leydig cells.

### 3.3. Induction of Autophagy Attenuates High Glucose-Induced Oxidative Stress in Leydig Cells

**Figure 4:**
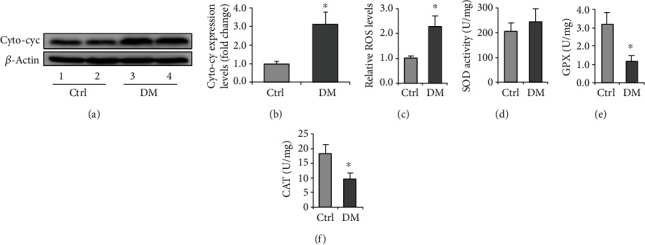
Expression of oxidative stress-related marker proteins in the diabetic testis and Leydig cells. (a) Representative immunoblotting of Cyto-cyc in the testis of diabetic mice. (b) Densitometric qualification of Cyto-cyc in the testis of diabetic mice. (c) Relative ROS levels in the testis of diabetic mice. (d) SOD activity in the testis of diabetic mice. (e) GPX content in the testis of diabetic mice. (f) CAT content in the testis of diabetic mice. DM: diabetes mellitus; Rap: rapamycin; HG: high glucose. *P* < 0.05 was considered to indicate a statistically significant difference. ^∗^*P* < 0.05 vs. Ctrl.

**Figure 5:**
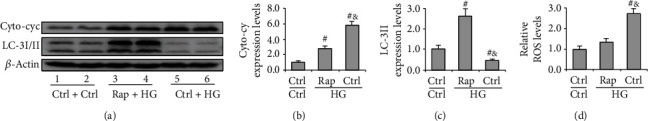
Expression of Cyto-cyc and LC-3 proteins in the diabetic testis and Leydig cells. (a) Representative immunoblotting of Cyto-cyc and LC-3I/II in Leydig cells treated with Rap and HG. (b) Densitometric qualification of Cyto-cyc in Leydig cells treated with Rap and HG. (c) Densitometric qualification of LC-3II in Leydig cells treated with Rap and HG. (d) Relative ROS levels in Leydig cells treated with Rap and HG. DM: diabetes mellitus; Rap: rapamycin; HG: high glucose. *P* < 0.05 was considered to indicate a statistically significant difference. ^#^*P* < 0.05 vs. Ctrl+Ctrl. ^&^*P* < 0.05 vs. Rap+HG.

Considering the significant apoptosis and autophagy observed in Leydig cells of the diabetic testes, we wondered how autophagy plays a role in Leydig cells' survival. Because autophagy is tightly associated with mitochondrial homeostasis and oxidative stress, we detected the level of cytochrome C in the cytoplasm (Cyto-cyc) of Leydig cells. When compared with that of the control, an increase in Cyto-cyc content was observed in diabetic mice (Figures 4(a) and 4(b)), accompanied by the increase of ROS (Figure 4(c)) and the mitigation of GPX and CAT (Figures 4(e) and 4(f)). However, we did not observe the palpable change of SOD in Leydig cells (Figure 4(d)). *In vitro*, we found that high glucose (HG) can induce the upregulation of Cyto-cyc (Figures 5(a) and 5(b)) and ameliorate LC-3II (Figures 5(a) and 5(c)). Nevertheless, rapamycin, an autophagy inducer, partially deregulated the effect of HG on Leydig cells (Figures 5(a) and 5(c)). Furthermore, we found that HG decreased cell viability, whereas rapamycin partially protected the cells (Supplementary Figure 3A). Meanwhile, the induction of autophagy attenuated the increase of ROS induced by HG (Figures 5(a) and 5(d)). These findings suggested that HG is implicated in the oxidative stress of Leydig cells via inhibition of autophagy, and induction of autophagy can partially rescue this phenotype.

### 3.4. HIF-1*α* /BNIP3 Pathway May Be Involved in the Autophagy Regulation in Sertoli Cells

**Figure 6:**
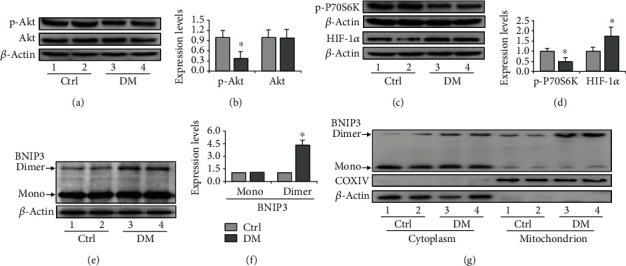
Activation of HIF-1*α* pathway in Sertoli cells of diabetic mice. (a) Representative immunoblotting of Akt and p-Akt in the testis of diabetic mice. (b) Densitometric qualification of Akt and p-Akt in the testis of diabetic mice. (c) Representative immunoblotting of p-P70S6K and HIF-1*α* in the testis of diabetic mice. (d) Densitometric qualification of p-P70S6K and HIF-1*α* in the testis of diabetic mice. (e) Representative immunoblotting of dimer and monomer BNIP3 in the testis of diabetic mice. (f) Densitometric qualification of dimer and monomer BNIP3 in the testis of diabetic mice. (g) Representative immunoblotting of dimer and monomer BNIP3 in the cytoplasm and mitochondrion. DM: diabetes mellitus. *P* < 0.05 was considered to indicate a statistically significant difference. ^∗^*P* < 0.05 vs. Ctrl.

Considering the divergent phenotypes in terms of autophagy regulation in Leydig and Sertoli cells, we explored the potential regulation of autophagy induction in Sertoli cells. Therefore, the levels of the predominant autophagy regulator p-Akt and its downstream effector p-P70S6K were detected in Sertoli cells. The results indicated the concomitant upregulation of both proteins (Figures 6(a)–6(d)), suggesting that the Akt pathway may be involved in autophagy regulation in Sertoli cells. HIF-1*α* is essential in autophagy regulation, especially under hypoxic or oxidative contexts. We showed that, similar to many other diabetic organs, diabetes increased the expression of HIF-1*α* in Sertoli cells (Figures 6(c) and 6(d)). Interestingly, even though diabetic mice exhibited significant formation of BNIP3 homodimer (Figures 6(e) and 6(f)), we did not observe the concomitant upregulation of BNIP3 monomer (Figures 6(e) and 6(f)). Because BNIP3 homodimerization is an important step for mitophagy initiation, we separated the mitochondrial component and detected the expression changes of both dimer and monomer forms of BNIP3 (Figure 6(g)). Both cytoplasmic and mitochondrial components consistently showed increased homo-BNIP3 (Figures 5(g) and 5(h)), while mono-BNIP3 remained unchanged in the cytoplasm (Figure 5(g)). These findings suggested that HIF-1*α* may be involved in mitophagy regulation by promoting the formation of BNIP3 homodimerization.

### 3.5. AGEs Promote Oxidative Stress in Sertoli Cells by Enhancing HIF-1*α*-Mediated Autophagy

**Figure 7:**
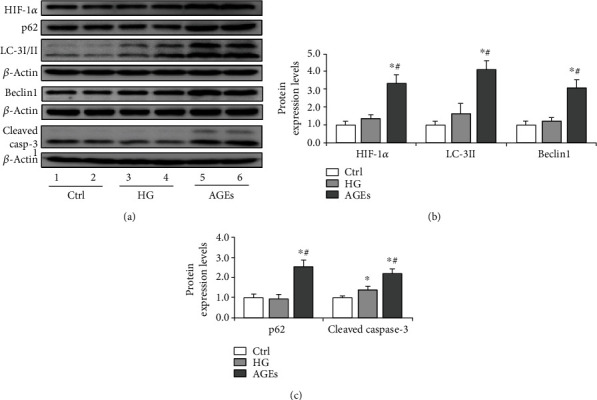
Expression of HIF-1*α*, LC-3I/II, p62, and cleaved caspase-3 during AGE-induced oxidative stress in Sertoli cells. Sertoli cells were treated with HG (20 mM) or AGEs (100 *μ*g/ml) for 72 h. AGEs aggravated HIF-1*α* expression and induced autophagy dysregulation. (a) Representative immunoblotting of HIF-1*α*, LC-3I/II, p62, Beclin1, and cleaved caspase-3 in Sertoli cells treated with HG and AGEs. (b) Densitometric qualification of HIF-1*α*, LC-3II, and Beclin1 in Sertoli cells treated with HG and AGEs. (c) Densitometric qualification of p62 and cleaved caspase-3 in Sertoli cells treated with HG and AGEs. *P* < 0.05 was considered to indicate a statistically significant difference. ^∗^*P* < 0.05 vs. Ctrl. ^#^*P* < 0.05 vs. HG.

**Figure 8:**
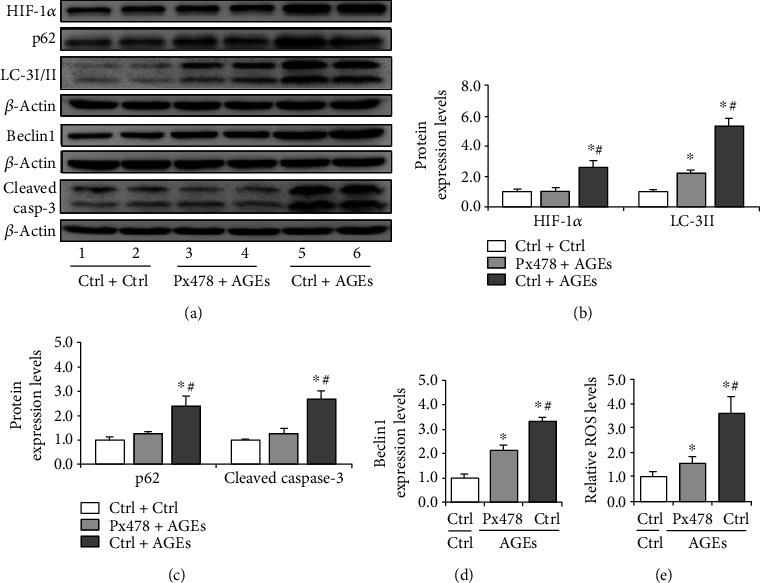
Effect of Px478 on HIF-1*α*, LC-3I/II, p62, and cleaved caspase-3 expression during AGE-induced oxidative stress in Sertoli cells. The cells were treated with AGEs (100 *μ*g/ml) for 72 h, and Px478 (20 *μ*M) was added 16 h before harvesting. (a) Representative immunoblotting of HIF-1*α*, LC-3I/II, p62, Beclin1, and cleaved caspase-3 in Sertoli cells treated with Px478 and AGEs. (b) Densitometric qualification of HIF-1*α*, LC-3II, and Beclin1 in Sertoli cells treated with Px478 and AGEs. (c) Densitometric qualification of p62 and cleaved caspase-3 in Sertoli cells treated with Px478 and AGEs. (d) Relative ROS levels in Sertoli cells treated with Px478 and AGEs. *P* < 0.05 was considered to indicate a statistically significant difference. ^∗^*P* < 0.05, vs. Ctrl+Ctrl. ^#^*P* < 0.05, vs. Px478+AGEs.

To explore the causative factors for autophagy induction, we treated Sertoli cells with HG and AGEs, a nonenzymatic product of glucose and proteins. The results indicated that the effects of HG on HIF-1*α*, LC-3II, and p62 expression were not apparent (Figures 7(a)–7(c)). However, AGEs significantly intensified the expression of HIF-1*α*, LC-3II, p62, and Beclin1 (Figures 7(a)–7(c)), suggesting a positive role of AGEs in autophagy regulation. The increase of p62 indicated a decrease in autophagy flux in Sertoli cells. Furthermore, Chloroquine, an autophagy inhibitor, partially abolished the effect of AGEs on Sertoli cells, and the expression of HIF-1*α* was also relatively reduced (Supplementary Figure 4). Moreover, we found that AGEs decreased cell viability while chloroquine partially protected cells (Supplementary Figure 3B). To further confirm the role of HIF-1*α* in AGE-induced autophagy, we treated the cells with Px478 to inhibit HIF-1*α* expression and observed the concomitant decrease of LC-3II (Figures 8(a) and 8(b)), suggesting a positive role for HIF-1*α* in autophagy regulation.

We also detected the effects of HG and AGEs on the apoptosis of Sertoli cells (Figures 7(a) and 7(c)). Comparatively, AGEs led to an apparent increase of cleaved caspase-3 expression (Figures 7(a) and 7(c)), while this effect could be partially reversed by HIF-1*α* inhibition (Figures 8(a), 8(c), and 8(d)). When measuring the level of ROS, we observed concomitant mitigation after Px478 treatment (Figure 8(e)). These results suggested that AGEs significantly contribute to enhanced autophagy levels and aggravate oxidative stress in Sertoli cells.

### 3.6. Upregulation of HIF-1*α* Ameliorates Testosterone Production in Leydig Cells

**Figure 9:**
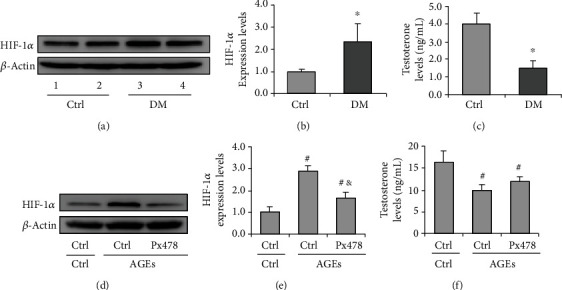
Expression of HIF-1*α* in the diabetic testis and Leydig cells. Leydig cells were treated with AGEs (100 *μ*g/ml) for 72 h, and Px478 was added 16 h before harvesting. (a) Representative immunoblotting of HIF-1*α* in the testis of diabetic mice. (b) Densitometric qualification of HIF-1*α* in the testis of diabetic mice. (c) Serum testosterone level in diabetic mice. (d) Representative immunoblotting of HIF-1*α* in Leydig cells treated with Px478 and AGEs. (e) Densitometric qualification of HIF-1*α* in Leydig cells treated with Px478 and AGEs. (f) Testosterone level in supernatant of Leydig cells treated with Px478 and AGEs. *P* < 0.05 was considered to indicate a statistically significant difference. ^∗^*P* < 0.05 vs. Ctrl. ^#^*P* < 0.05 vs. Ctrl+Ctrl. ^&^*P* < 0.05, vs. Px478+AGEs.

A previous study demonstrated the stable expression of HIF-1*α* in Leydig cells and its function as a crucial regulator of steroidogenesis [33]. However, how HIF-1*α* is regulated and its role in testosterone production remains unknown. In the present study, an improvement of HIF-1*α* content was detected in Leydig cells of diabetic mice (Figures 9(a) and 9(b)). Additionally, AGEs enabled the upregulation of HIF-1*α*, indicating a potential regulatory effect on HIF-1*α* expression (Figures 9(d) and 9(e)). Consistent with preceding results, diabetic mice exhibited downregulation of serum testosterone content (Figure 9(c)), and this effect could be recapitulated by AGEs treatment *in vitro* (Figure 9(f)). However, inhibition of HIF-1*α* attenuated the decrease in testosterone production (Figure 9(f)). These findings suggested that AGEs can induce HIF-1*α* expression, and this enhanced HIF-1*α* expression is associated with lowered testosterone levels.

## 4. Discussion

In this study, we investigated the role of autophagy in diabetes-induced testicular impairment and demonstrated that diabetes disrupted testicular structure and exerted dual effects on autophagy regulation in Sertoli and Leydig cells. Nevertheless, the autophagy dysregulation in both cell types contributed to skewed-oxidative homeostasis. Our results also suggested that enhanced HIF-1*α* expression is a driving force for autophagy in Sertoli cells and promotes oxidative stress in Leydig cells in an autophagy-independent manner.

Diabetes is an important causative factor for hormonal dysregulation, testicular impairment, and male infertility. Sertoli and Leydig cells are the major components of the testis, and they are, respectively, involved in sperm maturation and testosterone production. Sertoli cells are also required to form BTB to prevent the invasion of extracellular material into the intratubular fluid. Previous investigations have demonstrated the deleterious effect of diabetes on the integrity of BTB [34]. Similarly, the diabetic context can drive the change of the ultrastructure of Leydig cells and facilitate cell apoptosis [35]. However, most of these studies adopted a streptozotocin-induced mouse model, which is different from the pathological process. In the present study, we constructed a diabetic model by HFD feeding of rats and observed the increase of cell apoptosis, the loss of testicular architecture, and the atrophy of the testes. These findings are consistent with those from streptozotocin-induced diabetic mice, which validated the role of diabetes in male infertility.

Although several lines of evidence have confirmed the effect of diabetes on testicular damage, the mechanism remains elusive. It has been reported that the disruption of the microvascular system induced by Akt downregulation is one of the impactors of testicular impairment, and this effect could be recapitulated by HG treatment [36]. Autophagy is a stress-driven, self-degradation mechanism that is initiated under unfavorable conditions. The dysregulation of autophagy is a hallmark of many human diseases, especially cancer [37] and degradative diseases, including Alzheimer's disease [38]. Predominantly, the essential contribution of autophagy in testicular function has also been evaluated in several publications. However, the pathological function of autophagy under diabetic conditions has not been investigated yet. In our study, through IF staining and Western blot of autophagy-related proteins and AO staining, we found that diabetes plays dimorphic roles in autophagy regulation, promoting autophagy in Sertoli cells while inhibiting autophagy in Leydig cells. Nevertheless, both effects undermine cell functions.

Extensive research data suggest that ROS contribute to glucolipotoxicity in diabetes, leading to cellular and tissue dysfunction and damage [39]. Indeed, high ROS production, low ATP levels, and mitochondrial dysfunction are hallmarks of type 2 diabetes [40]. The increased oxidative stress has been confirmed in many diabetic tissues and detected in the testes [41, 42]. So far, compelling evidence has verified the decrease of testosterone in diabetic patients [43], and oxidative stress is detrimental to Leydig cell functions [44]. However, how diabetes drives oxidative stress and its phenotype has not been uncovered. Studies have shown that the overproduction of ROS caused by T1DM activates apoptotic signaling pathways in supporting cells, ultimately affecting the survival of these cells [45]. We mainly studied the effect of ROS excess caused by T2DM on Sertoli and Leydig cells. Our results indicated that diabetes induced an appreciable release of Cyto-cyc in Leydig cells, which was consistent with the increase of ROS and the downregulation of GPX and CAT, indicating mitochondrial balance and oxidative stress. The results also showed that HG is a causative factor for attenuated autophagy, and autophagy induction can attenuate the cytochrome C level in the cytoplasm, suggesting a protective role for autophagy in Leydig cell function under diabetic context.

The regulation of autophagy involves a spectrum of signaling pathways. Among these, Akt/mTOR mainly regulates autophagy under serum or glucose starvation. HIF-1*α* signaling is another pathway that could be activated under hypoxia or oxidative stress condition [46]. In the present study, we observed the amelioration of p-Akt expression and its downstream p-p70s6k in Sertoli cells. Conversely, we found that the level of HIF-1*α* was upregulated compared with that in the control. BNIP3 is a primary target of HIF-1*α* to regulate mitophagy in eukaryotes [47], which forms a homodimer to localize on mitochondria and label it for degradation. We found that the diabetic condition drives the formation of dimer-BNIP3 in the mitochondria of Sertoli cells, indicating a positive role for HIF-1*α*/BNIP3 pathway in autophagy regulation. Nonetheless, when treating Sertoli cells with HG, we did not observe a significant increase in autophagy level, while AGE treatment increased both HIF-1*α* and LC-3II expression. We also compared the effects of HG and AGEs on the apoptosis of Sertoli cells and found that AGEs can exacerbate caspase-3 cleavage compared to HG. This effect could be partially rescued by HIF-1*α* inhibition, suggesting that AGEs may regulate autophagy in Sertoli cells by promoting HIF-1*α* expression. We also showed that AGEs could induce HIF-1*α* expression in Leydig cells, which is related to attenuated testosterone levels.

## 5. Conclusion

Our present study showed a dual effect of diabetes on autophagy in Sertoli and Leydig cells, which drives the formation of oxidative stress in both cell types. HIF-1*α* plays a consistent role in exacerbating oxidative homeostasis in an autophagy-dependent or independent manner. Furthermore, the HFD-fed diabetic mouse model used in the present experiment was more in line with the pathological process of type 2 diabetes and is more helpful for studying type 2 diabetes. Our findings also draw attention to testicular damage in diabetic patients and indicate that HIF-1*α* inhibitors may become a new direction for research and treatment of diabetes. Although we have confirmed that the HIF-1*α* signaling plays a role in testicular injury in diabetic mice, further studies are still needed to identify possible HIF-1*α* upstream-regulated genes and their downstream target genes to explore whether it regulates or coregulates diabetes-induced autophagy leading to testicular damage.

## Data Availability

The original contributions presented in the study are included in the article, and further inquiries can be directed to the corresponding authors.

## Supplementary Materials

Supplementary Materials Figure S1: identification of Sertoli and Leydig cells by cell-specific marker expression. Figure S2: evaluation of changes of autophagy in the testis of diabetic mice by acridine orange staining. Figure S3: effects of autophagy on the cell viability of Sertoli and Leydig cells. Figure S4: effects of chloroquine on the expressions of HIF-1*α*, LC-3I/II, p62, and cleaved caspase-3 in Sertoli cells. Table S1: antibody information for Western blotting.
