# Supplementary material for: Diabetes-Induced Autophagy Dysregulation Engenders Testicular Impairment via Oxidative Stress
Source: Oxid Med Cell Longev. 2023 Feb 3;2023:4365895. doi: 10.1155/2023/4365895 (PMC9918358; doi:10.1155/2023/4365895)
Supplement: Supplementary Materials — Figure S1: identification of Sertoli and Leydig cells by cell-specific marker expression. Figure S2: evaluation of changes of autophagy in the testis of diabetic mice by acridine orange staining. Figure S3: effects of autophagy on the cell viability of Sertoli and Leydig cells. Figure S4: effects of chloroquine on the expressions of HIF-1α, LC-3I/II, p62, and cleaved caspase-3 in Sertoli cells. Table S1: antibody information for Western blotting. [file 4365895.f1.doc]

Journal: Oxidative Medicine and Cellular Longevity

Title: Diabetes-Induced Autophagy Dysregulation Engenders Testicular Impairment by Oxidative Stress

Authors: Renfeng Xu, Fan Wang, Zhenghong Zhang, Yan Zhang, Yedong Tang, Jingjing Bi, Congjian Shi, Defan Wang, Hongqin Yang, Zhengchao Wang, Zonghao Tang

**Supplementary Materials**

**Supplementary Figure 1**


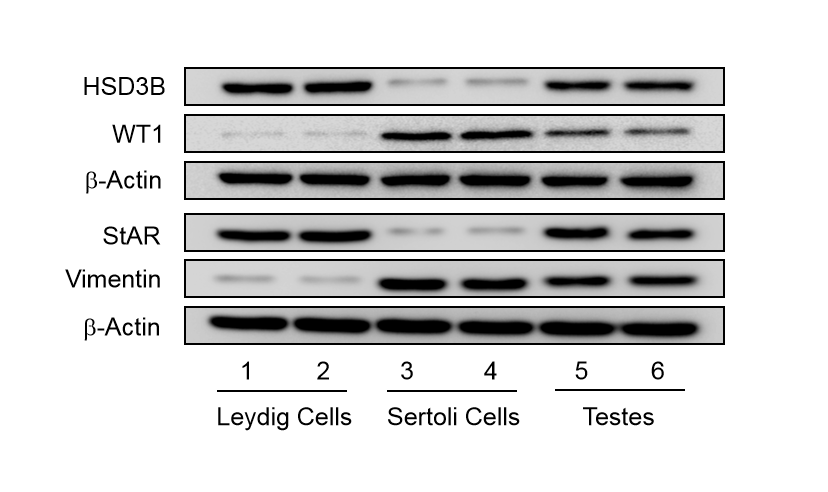


**Figure S1 Identification of Sertoli and Leydig cells by cell-specific marker expressions.** According to the previous reports, HSD3B and StAR were selected as the the markers of Leydig cell-specific expressions, while WT1and Vimentin were selected as the the markers of Sertoli cell-specific expressions. The samples were prepared and incubated with primary antibodies against HSD3B and StAR (Leydig cell-specific marker proteins) and against WT1and Vimentin (Sertoli cell-specific marker proteins), respectively, and then incubated with horseradish peroxidase-labeled goat anti-rabbit or mouse IgG. Eventually, the bands were visualized by using an ECL kit .

**Supplementary Figure 2**


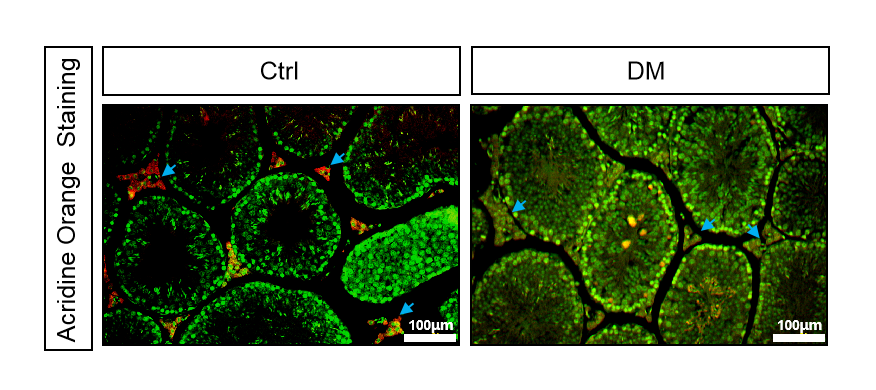


**Figure S2 Evaluation of changes of autophagy in the testis of diabetic mice by acridine orange staining.** After deparaffinization and hydration, the paraffin sections of testis were washed with PBS, and then AO staining solution was added dropwise and incubated in the dark for 30 min. After washing with PBS, the sections were mounted. Blue arrow indicates Leydig cells. DM: diabetes mellitus. Bar=100μm.

**Supplementary Figure 3**


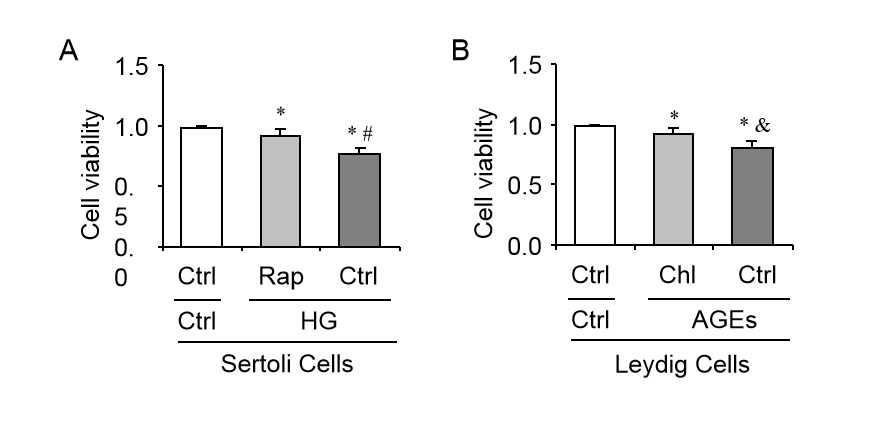


**Figure S3 Effects of autophagy on the cell viability of Sertoli and Leydig cells.** The Leydig cells were treated with AGEs for 72h, Chl was added 16h before harvesting. While the Sertoli cells were treated with Rap and HG. The cell viability of Sertoli and Leydig cells after 72h treatment was detected by the Cytometry Kit-8 (CCK8) assay for measuring viable cell numbers. After aspirating the used medium, add 20 μl of CCK8 solution and 200 μl of fresh medium to each well, and incubate at 37^o^C for 1h in the dark. Then read at 450 nm using a microplate reader. A: The cell viability of Sertoli cells treated with Rap and HG. B: The cell viability of Leydig cells treated with Chl and AGEs. Rap: Rapamycin. HG: high glucose. Chl: Chloroquine, an autophagy inhibitor. *P<0.05* was considered to indicate a statistically significant difference. *: *P<0.05*, vs. Ctrl + Ctrl. #: *P<0.05*, vs. Rap+ HG. &: *P<0.05*, vs. Chl + AGEs.

**Supplementary Figure 4**


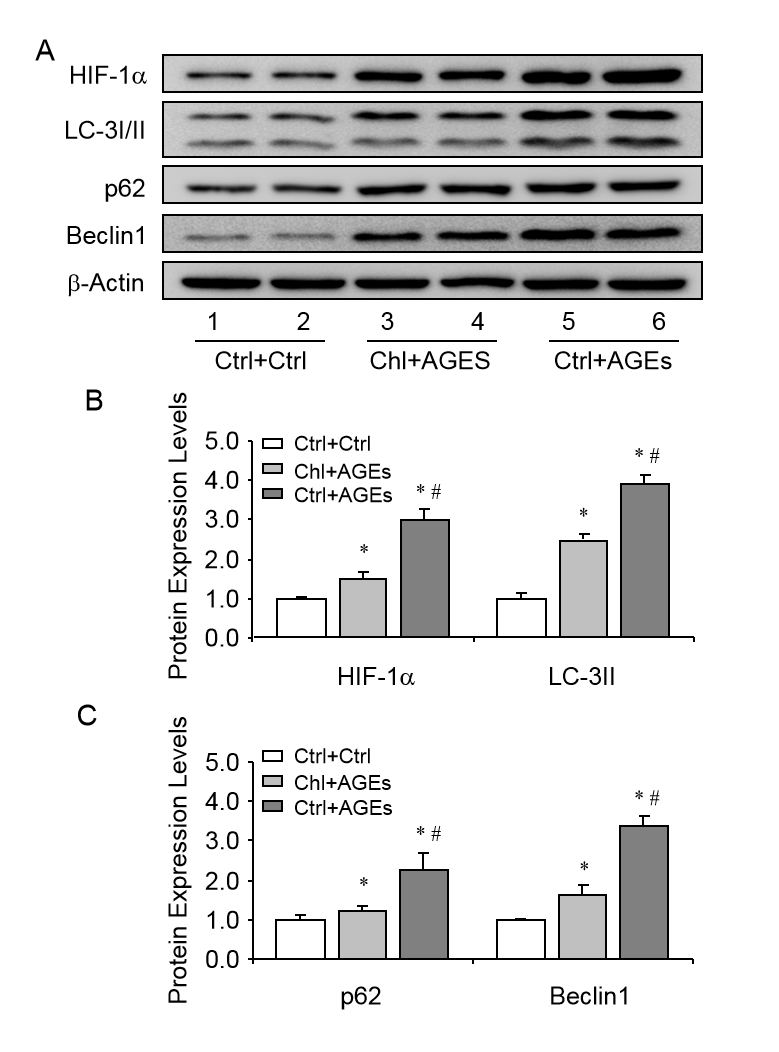


**Figure S4 Effects of chloroquine on the expressions of HIF-1α, LC-3I/II, p62 and Beclin1 in Sertoli cells**. The cells were treated with AGEs for 72h, Chl was added 16h before harvesting. A: Representative immunoblottling of HIF-1α, LC-3I/II, p62 and Beclin1 in Sertoli cells treated with Chl and AGEs. B: Densitometric qualification of HIF-1α and LC-3I/II. C: Densitometric qualification of p62 and Beclin1. Chl: Chloroquine, an autophagy inhibitor. *P<0.05* was considered to indicate a statistically significant difference. *: *P<0.05*, vs. Ctrl + Ctrl. #: *P<0.05*, vs. Chl+ AGEs.

**Supplementary Table 1**

**Table S1 Antibody informations for western blotting**

| Antibody Name | Company and City | Dilution Degree |
| --- | --- | --- |
| cleaved caspase-3 | Cell Signaling Technology, Boston, MA, USA | 1:1000 |
| β-actin | Protein Tech Group, Wuhan, China | 1:5000 |
| LC-3I/II | Abcam, Cambridge, MA, USA | 1:1000 |
| p62 | Abcam, Cambridge, MA, USA | 1:1000 |
| Beclin1 | Protein Tech Group, Wuhan, China | 1:2000 |
| anti-cytochrome C | Gene Tex, San Antonio, Texas, USA | 1:2000 |
| p-Akt | Protein Tech Group, Wuhan, China | 1:4000 |
| Akt | Protein Tech Group, Wuhan, China | 1:2000 |
| p-P70S6K | Cell Signaling Technology, Boston, MA, USA | 1:1000 |
| HIF-1α | Gene Tex, San Antonio, Texas, USA | 1:1000 |
| BNIP3 | Abcam, Cambridge, MA, USA | 1:1000 |
| COXIV | Protein Tech Group, Wuhan, China | 1:2000 |
| Goat anti-Mouse IgG | Beyotime Institute of Biotechnology, Haimen, China | 1:5000 |
| Goat anti-Rabbit IgG | Beyotime Institute of Biotechnology, Haimen, China | 1:5000 |
